# Dominant negative effect as a novel mechanism of SPAST gene mutation in a large family with hereditary spastic paraplegia

**DOI:** 10.1016/j.gendis.2023.101152

**Published:** 2023-10-27

**Authors:** Ke Deng, Haibo Ruan, Feifei Yu, Zhenle Pei, Congjian Xu, Shuo Zhang

**Affiliations:** a Shanghai Ji Ai Genetics & IVF Institute, Obstetrics and Gynecology Hospital of Fudan University, Shanghai 200000, China; b Shanghai Key Laboratory of Female Reproductive Endocrine Related Diseases, Shanghai 200000, China; c The First People's Hospital of Wenling, Wenling, Zhejiang 317500, China; d Rushan Hospital of Traditional Chinese Medicine, Rushan, Shandong 264599, China; e Department of Obstetrics and Gynecology, Fudan University, Shanghai 200000, China

Hereditary spastic paraplegia (HSP) is a group of disorders with genetic heterogeneity, lower-extremity weakness, and spasticity are the most common signs and symptoms. *SPAST* is the most frequently disease-causing gene. Spastin is a microtubule-severing enzyme encoded by SPAST that cleaves long microtubules into short fragments by interacting with other proteins or membranes. Hereditary spastic paraplegia type 4 (SPG4), which is autosomal dominant, is the clinical subtype associated with SPAST mutations. SPG4 accounts for up to one-third of all HSP cases and usually presents with isolated lower extremity spasticity, with or without bladder or sensory dysfunction.^1^ The main cause of SPG4 is believed to be spastin haploinsufficiency, which results from mutations in the SPAST gene.^2^ These mutations cause reduced spastin severing ability and insufficient microtubule cutting.^2^ However, haploinsufficiency alone cannot explain some phenomena in SPG4 models.^3^ Gain-of-function mutations contribute to the onsets of HSP, and truncated spastin may cause damage to the central nervous system tracts through an isoform-specific toxic effect.^4^ Furthermore, evidence that spastin forms oligomers and the discovery of cellular experiments suggest a possible dominant-negative effect. Here, we report a new SPAST mutation in clinical patients with a novel disease-causing mechanism.

In this study, we evaluated a large pedigree of SPG, including seven patients (three males and four females). Spastic paraplegia was inherited across at least four generations in this family (Fig. 1A). The proband (III-2), a 57-year-old man, presented to our hospital's outpatient clinic due to progressive difficulty walking caused by moderate spasticity of the lower limbs for the previous 50 years. He had an unknown gait disorder at seven years of age. This gait disorder had worsened in recent years, and he needed assistance from a walking aid or support. On neurological examination, the patient had a spastic gait. Increased deep tendon reflexes and Babinski signs were observed bilaterally. Sphincter dysfunction was one of the symptoms. The patients had mild cognitive impairment and delayed language development. Upper limb muscle strength was normal, while lower limb extensors and flexors were 3/5. Other affected family members had similar clinical features (Fig. 1B). Table S1 summarizes the clinical characteristics of all affected individuals.

**Figure 1:**
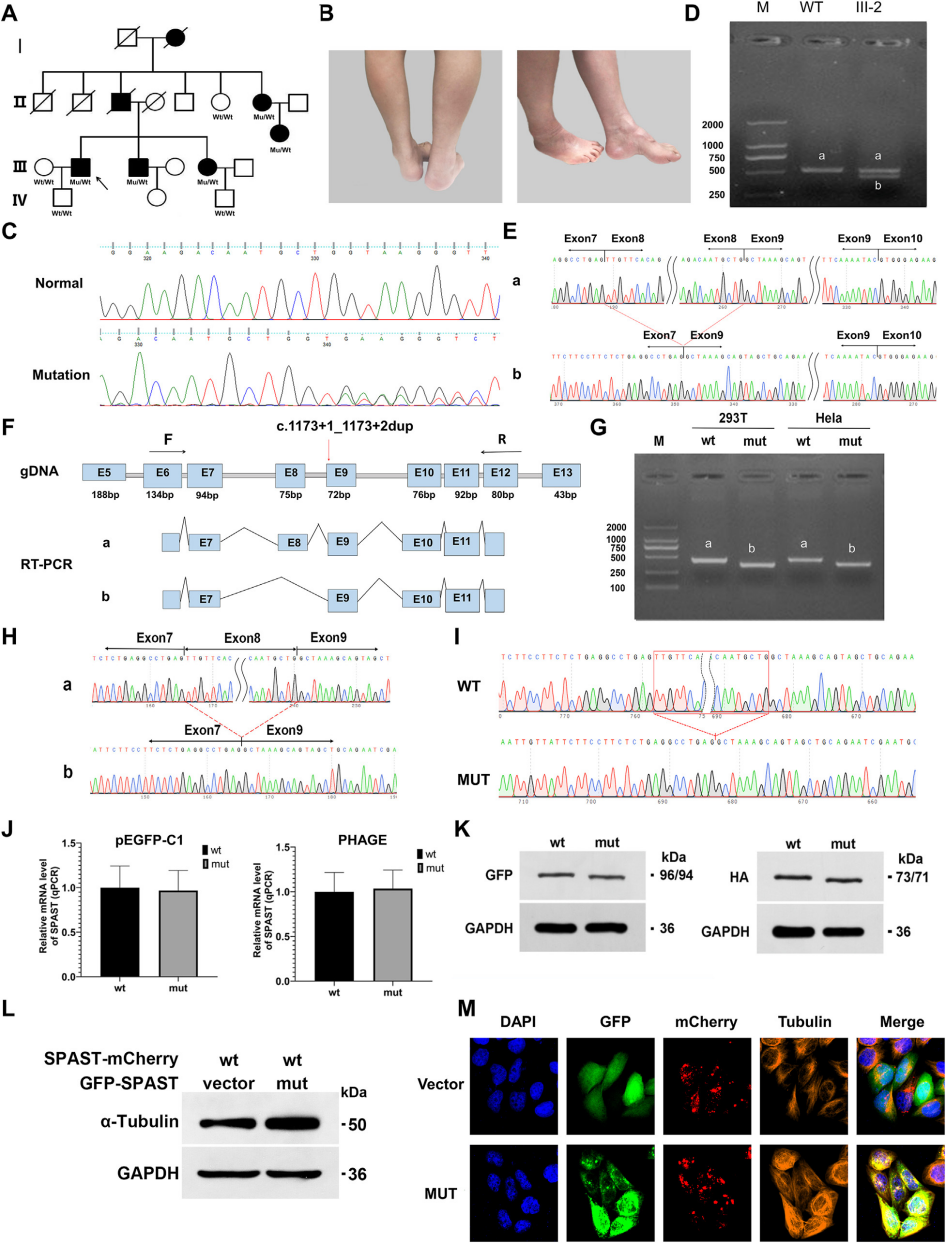
Dominant negative effect as a novel mechanism of SPAST gene mutation in a large family with hereditary spastic paraparesis (HSP). **(A)** Pedigrees of the HSP-affected families are shown. The affected male and female individuals are indicated with filled squares and circles, respectively. Normal individuals are shown as empty symbols. The proband is indicated with an arrow. Patients included I-2, II-3, II-7, III-2, III-3, III-5, and III-7, all of whom had similar symptoms of lower extremity spasticity. **(B)** Photograph of the lower extremities of one affected individual (III:3) in the family. The patient had leg and foot deformities manifested by pes cavus and atrophy of lower limb muscles. **(C)** The novel mutation was confirmed by Sanger sequencing. Affected family members (II-7, III-3, III-5, and III-7) and one unaffected individual (IV-2) underwent singer sequencing. DNA sequencing results of an unaffected family member (upper panel) and an affected family member (lower panel) are shown. **(D)** RT-PCR using RNA isolated from the blood of the proband and one healthy control. The band of the wide type is named “a”, and the bands of the proband are named “a” and “b”. A longer amplicon of approximately 528 bp detected in the control subject corresponded to the expected band size. III-2 had two bands, indicating heterozygosity. The b-band is shorter than the a-band. **(E)** RT-PCR product sequencing results. Sequencing analysis of normal and short-sized bands confirmed the deletion of 75 nucleotides of exon 8 in the shorter fragment. **(F)** Schematic diagram of the primer design and the c.1173 + 1_1173+2dup variant related abnormal splicing. The asterisk indicates the location of the c.1173 + 1_1173+2dup variant. **(G)** The minigene build strategy for pcDAN3.1-SPAST-wt/mut was to insert partial exon 7 (94 bp)–partial intron 7 (495 bp)–exon 8 (75 bp)–intron 8 (1385 bp)–exon 9 (72 bp) into pcDNA3.1. PcDAN3.1-SPAST-wt and pcDAN3.1-SPAST-mut plasmids were transiently transfected into HeLa and 293T cells. RT-PCR of total RNA obtained from cells transfected with pcDAN3.1-SPAST-wt produced a 350-bp band presenting correct mRNA splicing, and a shorter band was observed in cells transfected with pcDAN3.1-SPAST-mut. **(H)** Mini-gene product sequencing results: (i) The wild-type mini-gene (pcDNA3.1-SPAST-wt) formed normal mRNA composed of exons 7, 8, and 9; (ii) The mutant mini-gene (pcDNA3.1-SPAST-mut) caused a splicing abnormality, resulting in the deletion of exon 8. **(I)** Sequencing results showed that the mutation c.1173 + 1_1173+2dup was successfully introduced. **(J)** The constructed recombinant eukaryotic expression vectors, phage-SPAST-wt/mut and pEGFP-C1-SPAST-wt/mut, were transiently transfected into 293T cells. The expression level of WT or mut mRNA in transfected 293T cells was detected by qPCR. There was no significant difference in mRNA expression. **(K)** The protein expression of wt-spastin and mut-spastin in 293T cells. Western blot analysis showed that the protein levels of the mut were similar to those of the wt. **(L)** Effects of mutation on microtubule stability. Tubulin acetylation of transfected cells was analyzed by Western blotting with the indicated antibodies. The expression of acetylated α-tubulin increased in the mCherry-SPAST-wt and pEGFP-SPAST-mut co-transfected groups compared with that in the mCherry-SPAST-wt and pEGFP co-transfected groups. **(M)** Effects of mutation on spastin microtubule-severing activity. Representative immunofluorescence images for mt-spastin (green), wt-spastin (red), α-tubulin (orange), and nuclei (blue) were shown. In the mCherry-SPAST-wt and pEGFP co-transfected groups, microtubule protein was severed, whereas, in the mCherry-SPAST-wt and pEGFP-SPAST-mut co-transfected groups, the microtubule-severing activity was significantly reduced. Spastin-labeled filaments in pEGFP-SPAST-mut-transfected cells colocalized with tubulin.

To identify the mutation site, we performed whole exome sequencing analysis of genomic DNA for the proband (III-2), his wife (III-1), and son (IV-1), and identified a novel mutation (c.1173 + 1_1173+2dup) at the splice site of *SPAST* in the proband. The proband's wife and son did not exhibit mutations. Sanger sequencing was conducted in other patients of this family (II-7, III-3, III-5, and III-7) and two healthy individuals (II-6, IV-3) to valid the mutation (Fig. 1C). We found the disease and the mutation co-segregated within the family. The mutation was present in all affected individuals while absent in unaffected individuals.

During evolution, the locations of splice donors and acceptors are highly conserved. Mutations in splice sites usually result in abnormal pre-mRNA splicing, leading to cryptic splice site activation, exon skipping, formation of pseudo-exons within introns, and intron retention. To assess whether the *SPAST* mutation affects precursor mRNA splicing, we performed RT-PCR using RNA samples extracted from the blood of the proband and one healthy control (Fig. 1F). The PCR products showed amplicons of two distinct lengths when they were electrophoresed on an agarose gel (Fig. 1D). The control participant had a single longer amplicon, spanning about 528 bp, which was in line with the anticipated band size. III-2 had two bands, indicating heterozygosity. Sequencing analysis of normal and short-sized bands confirmed the deletion of 75 nucleotides of exon 8 in the shorter fragment (Fig. 1E).

To further determine whether the mutation could affect the splicing of exon 7–exon 8–exon 9, we conducted an *in vitro* minigene splicing assay. The minigene for pcDAN3.1-SPAST-wt/mut was constructed by inserting partial exon 7 (94 bp)–partial intron 7 (495 bp)–exon 8 (75 bp)–intron 8 (1385 bp)–exon 9 (72 bp) into pcDNA3.1. To determine whether the mutation would have an impact on the splicing of exon 7–exon 8–exon 9, pcDAN3.1-SPAST-wt and pcDAN3.1-SPAST-mut plasmids were transiently transfected into HeLa and 293T cells. RNA was isolated, RT was performed, and then the product was separated on 2 % agarose gel. Total RNA product from cells transfected with pcDAN3.1-SPAST-wt produced a 350-bp band indicating normal mRNA splicing, but cells transfected with pcDAN3.1-SPAST-mut produced a shorter band (Fig. 1G). Sequencing analysis of RT-PCR products revealed that the SPAST donor splice-site mutation resulted in the loss of exon 8 of SPAST during mRNA splicing (Fig. 1H). Sequencing results confirmed the target fragment of pcDNA3.1-SPAST-wt and mut (Fig. S1A). A schematic diagram of mini-gene construction is shown in Figure S1B. Meanwhile, the minigene for pcMINI-SPAST-wt/mut got consistent results (Fig. S2).

To determine the effect of the mutation on the expression of mRNA and protein levels, the constructed recombinant eukaryotic expression vectors, phage-SPAST-wt/mut and pEGFP-C1-SPAST-wt/mut, were transiently transfected into 293T cells (Fig. 1I). After 48 h, there was no significant difference in cell state between the wt- and mut-transfected groups and mut mRNA expression did not change significantly, demonstrating that the c.1173 + 1_1173+2dup mutation had no significant effect on RNA stability (Fig. 1J and Table S3, 4). Western blot examination revealed that the protein levels of the mut were similar to those of the wt (Fig. 1K).

Spastin is a microtubule-cleaving ATPase that separates longer microtubules into shorter ones, regulating microtubule quantity and motility as well as the distribution of their dynamic plus ends. Functional studies have revealed that the microtubule-binding domain and ATPase AAA domain located between residues 270–328 and 342–599, respectively, are sufficient for hexamerization and microtubule severing. The mutation in this study resulted in the deletion of amino acids 367–391 of SPAST. Amino acids 342–599 of this protein constitute an ATPase domain, a key region for binding and hydrolyzing ATP; therefore, the deletion of amino acids 367–391 may lead to the lack of enzymatic activity in SPAST. To explore the potential effect of mutation on spastin function and subcellular location of spastin, the recombinant eukaryotic expression vector mCherry-SPAST-wt was created and co-transfected into 293T cells with pEGFP or pEGFP-SPAST-mut. Microtubules in transfected 293T cells were stained with an anti-α-tubulin antibody (Table S5). Microtubule protein was severed in the mCherry-SPAST-wt and pEGFP co-transfected groups, whereas in the mCherry-SPAST-wt and pEGFP-SPAST-mut co-transfected groups, the microtubule-severing activity was significantly reduced (Fig. 1L, M). In addition, we found that spastin-labeled filaments in pEGFP-SPAST-mut-transfected cells colocalized with tubulin, indicating that the mut protein inhibited the function of the wt protein with a dominant-negative effect (Fig. 1M).

Tubulin acetylation is a marker of stable microtubules.^5^ Western blot analysis showed that the expression of acetylated α-tubulin increased in the mCherry-SPAST-wt and pEGFP-SPAST-mut co-transfected groups compared with that in the mCherry-SPAST-wt and pEGFP co-transfected groups, indicating that the mut protein increased microtubule stability and inhibited the severance of microtubules by the wt protein (Fig. 1L). The mut protein may form a hexamer with the wt protein and thereby interfere with its function.

In summary, our findings suggest that the mut SPAST protein may perform a dominant-negative effect, which is the first clinical pedigree to confirm truncated spastin that interferes with normal protein function, providing an alternative pathological mechanism for HSP. More studies are warranted to further elucidate the dominant-negative effect.

## Author contributions

Conceptualization: Shuo Zhang, Ke Deng, and Haibo Ruan; Sample collecting: Ke Deng, Haibo Ruan, and Feifei Yu; Writing draft: Ke Deng and Haibo Ruan; Data curation and statistics: Ke Deng, Haibo Ruan, Feifei Yu, and Zhenle Pei; Writing-review and editing: Ke Deng, Haibo Ruan, Zhenle Pei, Shuo Zhang, and Congjian Xu.

## Conflict of interests

The authors declare no conflict of interests.

## Funding

The research was supported by the Shanghai Science and Technology Innovation Action Plan Program of China (No. 20Y11907200, 22Y11907200), the 10.13039/501100001809National Natural Science Foundation of China (No. 82201807), the Municipal Human Resources Development Program for Outstanding Young Talents in Medical and Health Sciences in Shanghai, China (No. 2022YQ075), and Shanghai “Rising Stars of Medical Talents” Youth Development Program (China).

## Figures and Tables

**Figure:**
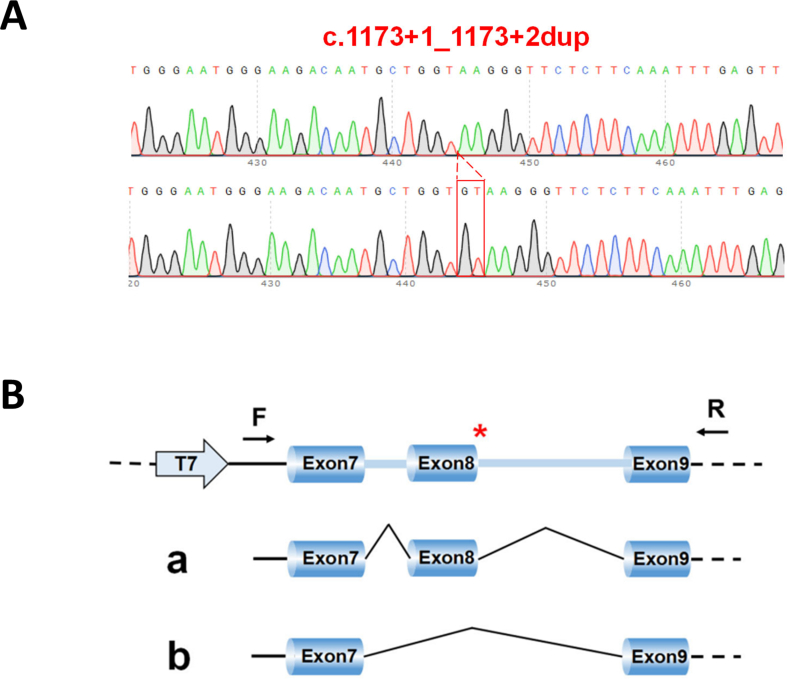
Figure S1 Results of the pcDNA3.1 vector mini-gene splicing assay. **(A)** Sequencing results of the target fragment of pcDNA3.1-SPAST-wt (upper panel) and mut (lower panel) mini-gene. **(B)** Schematic diagram of mini-gene construction and the c.1173 + 1_1173+2dup variant related abnormal splicing. The asterisk indicates the location of the c.1173 + 1_1173+2dup variant.

**Figure:**
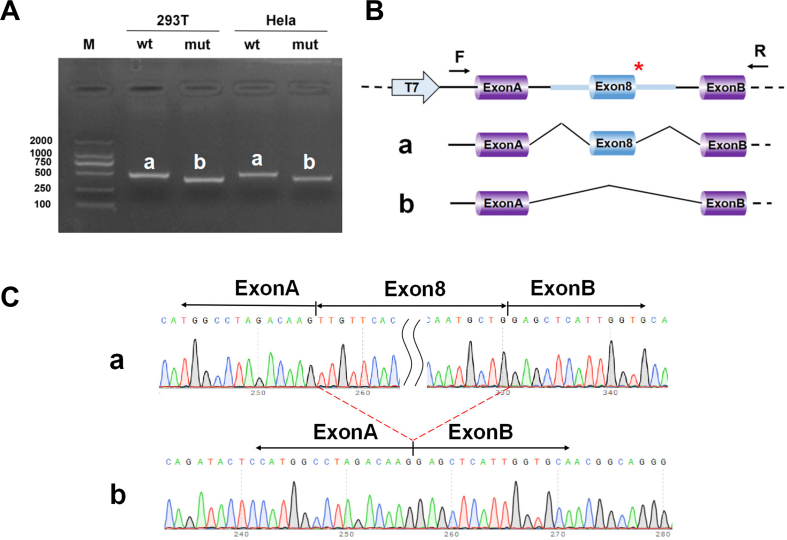
Figure S2 Results of the pcMINI vector mini-gene splicing assay. **(A)** Gel electrophoresis of RT-PCR products: the band of the wild-type was bigger than the mutant. **(B)** Schematic diagram of mini-gene construction and the c.1173 + 1_1173+2dup variant related abnormal splicing. The asterisk indicates the location of the c.1173 + 1_1173+2dup variant. **(C)** Mini-gene product sequencing results: (i) The wild-type mini-gene (pcMINI-SPAST-wt) formed normal mRNA composed of exons A, 8, and B; (ii) The mutant mini-gene (pcMINI-SPAST-mut) caused a splicing abnormality, resulting in the deletion of exon 8.
