# Supplementary material for: Dominant negative effect as a novel mechanism of SPAST gene mutation in a large family with hereditary spastic paraplegia
Source: Genes Dis. 2023 Oct 27;11(5):101152. doi: 10.1016/j.gendis.2023.101152 (PMC11176630; doi:10.1016/j.gendis.2023.101152)
Supplement: Multimedia component 1 [file mmc1.docx]

**Supplementary**

**Materials and Methods**

**T****otal exon detection and analysis**

For extraction of genomic DNA, we used the QIAamp DNA Blood Mini Kit (QIAGEN, Hilden, Germany) to extract from blood samples. The proband (III-2), his wife, and son (III-1 and IV-1) underwent Whole exome analysis (WES). NimbleGen SeqCap EZ Human Exome Library v2.0 (NimbleGen, Madison, WI, USA) was used to capture the exome, and was then sequenced on a HiSeq2000 platform (Illumina, San Diego, USA).

Using Sanger sequencing for other affected family members (II-7, III-3, III-5, and III-7) and one unaffected individual (IV-2), we found that the variant with the disease co-segregated in the pedigree (**Fig. 1C**). Primers were designed to amplify *SPAST* exons, including all sequences at exon-intron junctions (forward primer, 5'-GAAGCAGAGTTCCCCTATGTTGC-3' and reverse primer, 5'- AGACAGGTGTGGTGGCGTGTG-3').

**RNA isolation and real-time polymerase chain reaction (RT-PCR)**

Blood samples were obtained from the proband and healthy sex- and age-matched controls. RNA was isolated from blood samples using the Blood Total RNA Quick Extraction Kit (Bioteke, Beijing,China) according to the kit instructions. cDNA was reverse transcribed using the Reverse Transcription Kit (YESEN, Shanghai, China). PCR products were identified by agarose gel electrophoresis and confirmed with sequencing.  The oligonucleotide primers used for RT-PCR of *SPAST* were forward 5'—GGAATGTGGACAGCAACCTT–3' and reverse 5'—CATCAAGCTCTTGTGGCCTA–3'.

**Construction of pc minigene and transcription analysis**

Wild-type (wt) SPAST was obtained by nested PCR using genomic DNA as a template. The deletion mutation was introduced using a pair of primers. Mutant (mut) *SPAST* was amplified using overlap extension PCR. **Table S2** contains the primer sequences used in minigene assay.

The PCR products were digested with restriction enzymes before being ligated into the restriction sites of pcDNA3.1 (Bioeagle Biotech Company Ltd., Wuhan, China) and pcMINI to generate recombinant plasmids pcMINI- and pcDNA3.1-wt/mut **(Fig. S1B; Fig.S2C**). The recombinant plasmids were ligated overnight at 4°C and then transformed into competent *Escherichia coli* DH5α cells.After 12 hours, colony PCR was used to confirm the construction of the required base changes. To confirm the mutation, plasmids were sequenced (**Fig. S1A; Fig.S2A**). For plasmid extraction, a Rapid Plasmid Mini Kit (SIMGEN, Hangzhou, China) was used according to the manufacturer's instructions.

HEK-293T and HeLa cells were cultured. The recombinant plasmids were transiently transfected into HEK-293T and HeLa cells using Liposomal Transfection Reagent (SIMGEN, Hangzhou, China).

Total RNA was extracted after transfection, using an RNA extraction kit (TaKaRa, Dalian, China) using the TRIzol method. cDNA was prepared according to the manufacturer’s instructions using a reverse transcription kit (YESEN, Shanghai, China ). UV spectrophotometry was used to determine the concentration and purity of extracted RNA. 2% agarose gel electrophoresis was performed to identify the PCR products and verified by sequencing.

**Plasmid construction and transfection**

The full-length *SPAST* cDNA was synthesized, and phage-SPAST-wt fragment was obtained by PCR amplification using phage-SPAST-SalI-F and phage-SPAST-NotI-R as primers; pEGFP-C1-SPAST-HindIII-F and pEGFP-C1-SPAST-BamHI-R were used as primers to obtain the pEGFP-C1-SPAST-wt fragment; SPAST-MUT-F/ SPAST-MUT-R primers were used to obtain mut fragments (**Table S3**). SalI and NotI were used to insert wt and mut into the phage vectors; HindIII and BamHI were used to insert wt and mut into the pEGFP-C1 vector.

293T cells were cultured with DMEM culture medium containing 10% FBS. Lipofectamine 2000 (Thermo Fisher Scientific, Waltham, MA, USA) was used for transient transfection of cells. After 48 h of transfection, total cellular RNA and proteins were extracted and analyzed using RT-PCR and Western blotting.

**RT-PCR**

RNA from the samples was isolated using a Blood Total RNA Quick Extraction Kit (Bioteke, Beijing,China) and was reverse-transcribed using a reverse transcription kit (YESEN, Shanghai, China). RT-PCR was performed using SYBR Green Mix (YESEN, Shanghai, China). To identify the expression of SPAST-wt and SPAST-mut, primers were designed using two sets of vectors (**Table S4**).

**Western Blotting**

Total protein was extracted from cell. Protein concentration was determined using a BCA kit (Beyotime, Shanghai, China). Heat denatures the proteins, which are subsequently separated by electrophoresis on a 10% sodium dodecyl sulfate-polyacrylamide gel and transferred to a nitrocellulose membrane. After overnight incubation with the primary antibody at 4°C, the membranes were incubated with the secondary antibody at room temperature. (**Table S5**).

**Immunofluorescence**

Transfected cells were cultured for 24 hours, fixed with 10% formaldehyde for 15 minutes and treated with 0.5% Triton X-100 for toumo-permeable membranes. After blocking with 5% bovine serum albumin for 30 minutes , the cells were washed with PBS and incubated overnight at 4 °C with a primary antibody (**Table S5**), followed by incubation with a secondary antibody. DAPI was used to stain the nuclei. Confocal microscopy was used to take images.
