# Supplementary material for: Dominant negative effect as a novel mechanism of SPAST gene mutation in a large family with hereditary spastic paraplegia
Source: Genes Dis. 2023 Oct 27;11(5):101152. doi: 10.1016/j.gendis.2023.101152 (PMC11176630; doi:10.1016/j.gendis.2023.101152)
Supplement: Multimedia component 2 [file mmc2.docx]

**FIGURE LEGENDS**

**Figure 1** Dominant negative effect as a novel mechanism of SPAST gene mutation in a large family with HSP. **(A)** Pedigrees of the hereditary spastic paraparesis (HSP)-affected families are shown. The affected male and female individuals are indicated with filled squares and circles, respectively. Normal individuals are shown as empty symbols. The proband is indicated with an arrow. Patients included I-2, II-3, II-7, III-2, III-3, III-5, and III-7, all of whom had similar symptoms of lower extremity spasticity. **(B)** Photograph of the lower extremities of one affected individual (III:3 in Figure 1A) in the family. The patient had leg and foot deformities manifested by pes cavus and atrophy of lower limb muscles. **(C)** The novel mutation was confirmed by Sanger sequencing. Affected family members (II-7, III-3, III-5, and III-7) and one unaffected individual (IV-2) underwent singer sequencing. DNA sequencing results of an unaffected family member (upper panel) and an affected family member (lower panel). **(D)** RT-PCR using RNA isolated from blood of proband and one healthy control. The band of the wide-type is named a, the bands of the proband are named a and b. A longer amplicon of approximately 528 bp detected in the control subject corresponded to the expected band size. III-2 had two bands, indicating heterozygosity. The b-band is shorter than the a-band. **(E)** RT-PCR product sequencing results. Sequencing analysis of normal and short-sized bands confirmed the deletion of 75 nucleotides of exon 8 in the shorter fragment. **(F)** Schematic diagram of the primer design and the c.1173+1_1173+2dup variant related abnormal splicing. The asterisk indicates the location of the c.1173+1_1173+2dup variant. **(G)** The minigene build strategy for pcDAN3.1-SPAST-wt/mut was to insert partial exon 7 (94 bp)–partial intron 7(495 bp)–exon 8(75 bp）–intron 8 (1,385 bp)–exon 9 (72 bp）into pcDNA3.1. PcDAN3.1-SPAST-wt and pcDAN3.1-SPAST-mut plasmids were transiently transfected into HeLa and 293T cells. RT-PCR of total RNA obtained from cells transfected with pcDAN3.1-SPAST-wt produced a 350-bp band presenting correct mRNA splicing, and a shorter band was observed in cells transfected with pcDAN3.1-SPAST-mut. **(H)** Mini-gene product sequencing results: (a) The wild-type mini-gene (pcDNA3.1-SPAST-wt) formed normal mRNA composed of exons 7, 8 and 9; (b) The mutant mini-gene (pcDNA3.1-SPAST-mut) caused a splicing abnormality, resulting in the the deletion of exon 8. **(I)** Sequencing results showed that the mutation c.1173+1_1173+2dup was successfully introduced. **(J)** The constructed recombinant eukaryotic expression vectors, phage-SPAST-wt/mut and pEGFP-C1-SPAST-wt/mut, were transiently transfected into 293T cells. The expression level of WT or mut mRNA in transfected 293T cells was detected by qPCR. There was no significant difference in mRNA expression. **(K)** The protein expression of wt-spastin and mut-spastin in 293T cells. Western blot analysis showed that the protein levels of the mut were similar to those of the wt. **(L)** Effects of mutationon microtubule stability. Acetylation-Tubulin of transfected cells were analyzed by Western blotting with the indicated antibodies. The expression of acetylated α-tubulin increased in the mCherry-SPAST-wt and pEGFP-SPAST-mut co-transfected groups compared to that in the mCherry-SPAST-wt and pEGFP co-transfected groups. **(M)** Effects of mutation on spastin microtubule-severing activity. Representative immunofluorescence images for mt-spastin (green), wt-spastin(red),α-tubulin (orange), and nuclei (blue) were shown. In the mCherry-SPAST-wt and pEGFP co-transfected groups, microtubule protein was severed, whereas in the mCherry-SPAST-wt and pEGFP-SPAST-mut co-transfected groups, the microtubule-severing activity was significantly reduced. Spastin-labeled filaments in pEGFP-SPAST-mut-transfected cells colocalized with tubulin.

**Supplementary Figures**

**
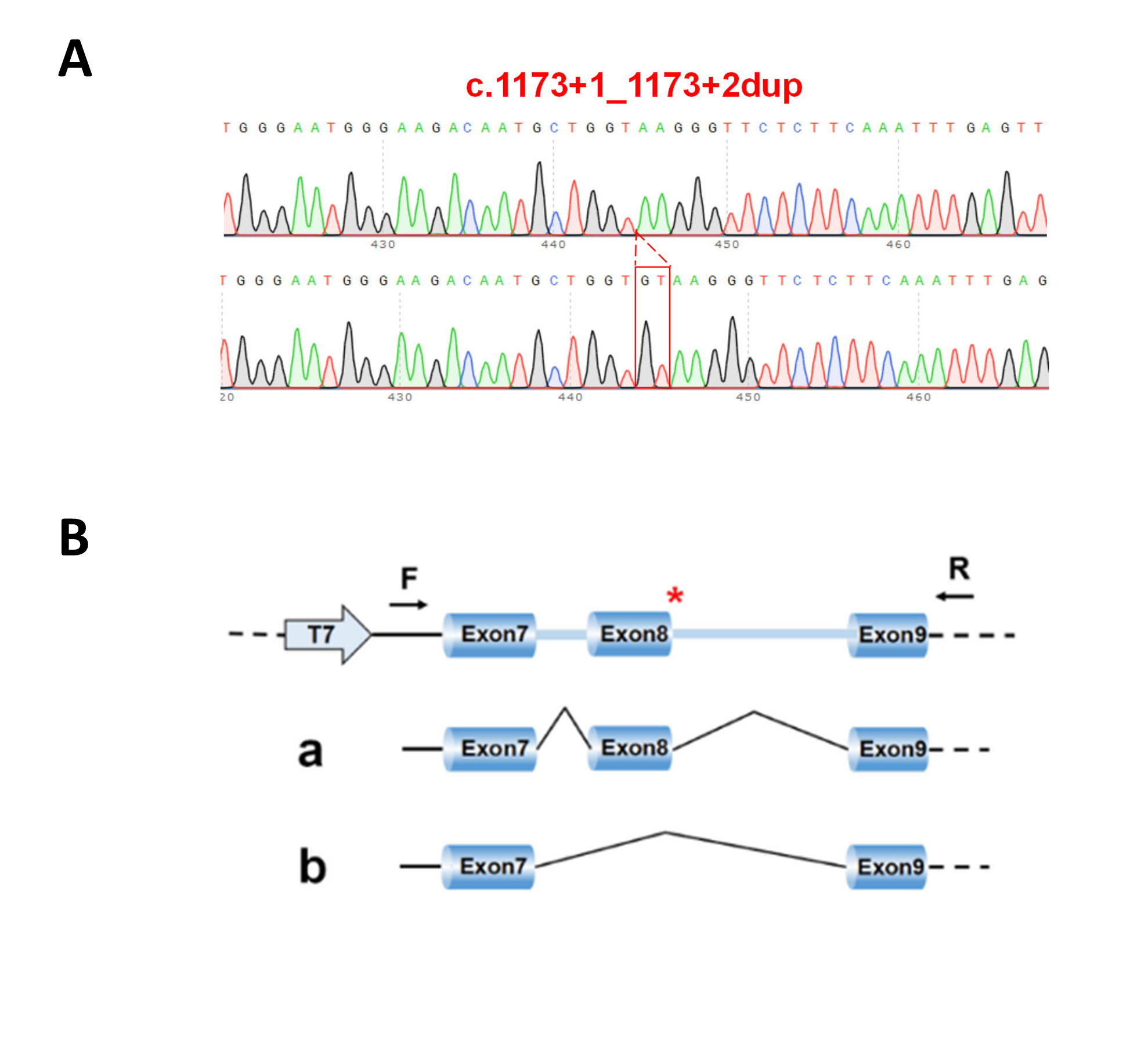
**

**Figure S1** Results of the pcDNA3.1 vector mini-gene splicing assay.**(A)** Sequencing results of the target fragment of pcDNA3.1-SPAST-wt (upper panel) and mut (lower panel) mini-gene. **(B)** Schematic diagram of mini-gene construction and the c.1173+1_1173+2dup variant related abnormal splicing. The asterisk indicates the location of the c.1173+1_1173+2dup variant.

**
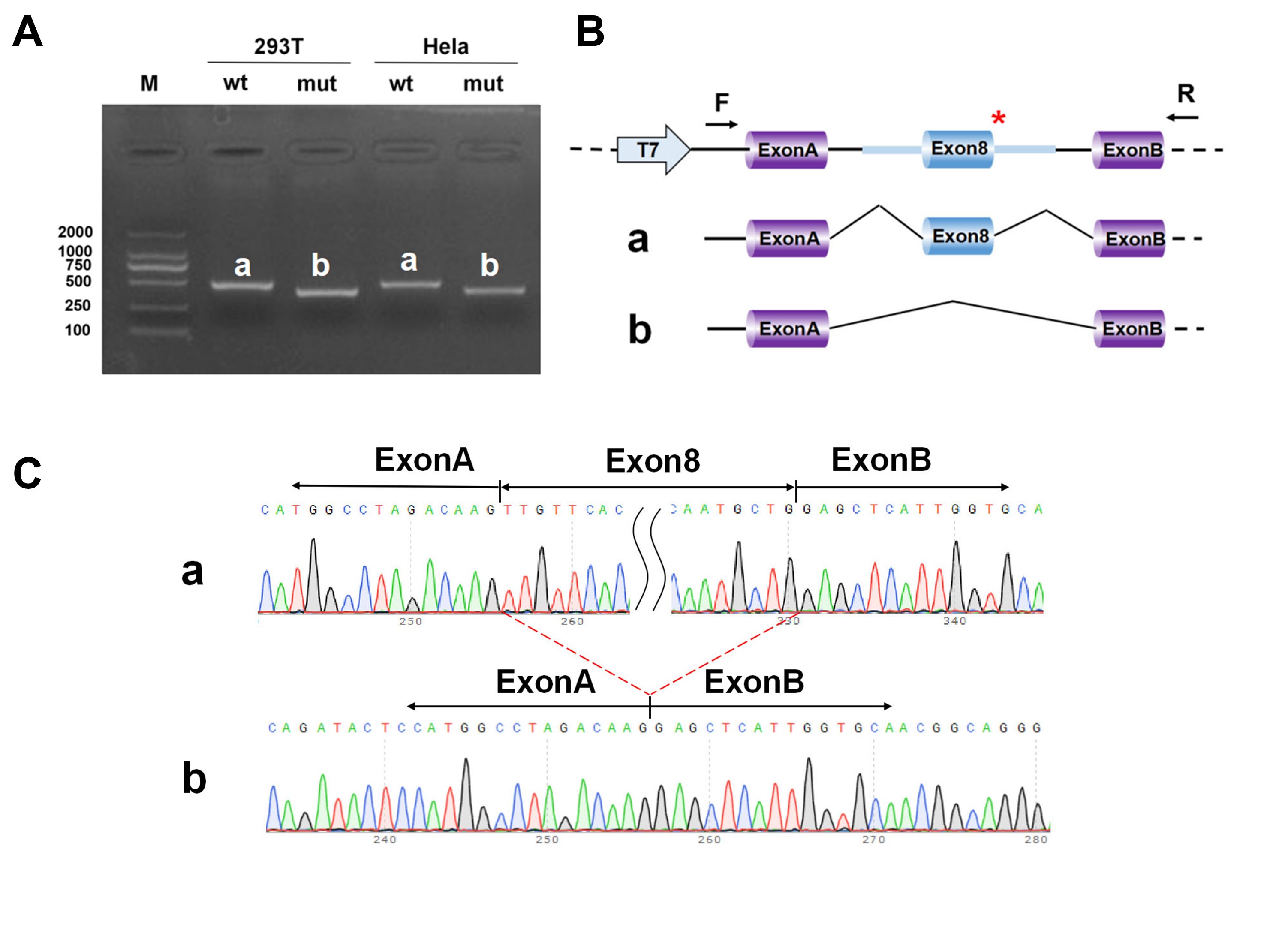
**

**Figure S2** Results of the pcMINI vector mini-gene splicing assay. **(A)** Gel electrophoresis of RT-PCR products: the band of the wild-type was bigger than the mutant. **(B)** Schematic diagram of mini-gene construction and the c.1173+1_1173+2dup variant related abnormal splicing. The asterisk indicates the location of the c.1173+1_1173+2dup variant. **(C)** Mini-gene product sequencing results: (a) The wild-type mini-gene (pcMINI-SPAST-wt ) formed normal mRNA composed of exons A, 8 and B; (b) The mutant mini-gene (pcMINI-SPAST-mut) caused a splicing abnormality, resulting in the the deletion of exon 8.

**Table S1 Clinical features of the affected individuals within the family**

| Individual ID | III-2 | III-5 | III-3 | III-7 | I-2 | II-3 |
| --- | --- | --- | --- | --- | --- | --- |
| Sex | M | F | M | F | F | M |
| Age | 57 | 59 | 55 | 53 | Died at 83 | Died at 74 |
| Age at onset (years)^a^ | 7 | 10s | 10s | 10s | na | na |
| Disease duration (years) | 50 | ＞45 | ＞40 | ＞40 | na | na |
| Disability stage ^b^ | 3 | 3 | 2 | 3 | 3 | 2 |
| Lower limb muscle force | 3 | 1 | 3 | 3 | na | na |
| Spasticity  UL/LL | -/+ | -/+ | -/+ | -/+ | na | na |
| Weakness  UL/LL | -/+ | -/+ | -/+ | -/+ | -/+ | -/+ |
| Atrophy  UL/LL | -/- | -/- | -/- | -/- | - | - |
| Resting tremor | - | - | + | - | na | na |
| Alopecia | - | + | - | - | na | na |
| Sensory deficits | - | - | - | - | na | na |
| Mental retardation | + | + | + | + | na | na |
| Hypopsia | - | - | - | - | na | na |
| Kayser-Fleischer ring | - | - | - | - | na | na |
| Nystagmus | - | - | - | - | na | na |
| Dysaudia | - | - | - | - | na | na |
| Urinary tract infection | slight | slight | slight | slight | na | na |
| Sphincter disturbances | + | + | + | + | na | na |
| Pes cavus | + | + | + | na | na | na |
| Ankle clonus | - | + | + | na | na | na |
| Babinski sign | + | + | + | na | na | na |
| Medical conditions complicating disability | Hepatitis;  Diabetes mellitus | na | na | na | na | Alzheimer's disease;  Cerebral thrombus |

+ and −, indicate the presence and absence of a feature, respectively.

a

Age at onset was calculated approximately as the time when difficulty in walking first appeared in the affected individuals.

b

Disability stages: 1, no mobility problems or slight stiffness of the legs; 2, moderate gait stiffness; 3, problems running, but able to walk alone; 4, problems walking; 5, wheelchair user.

**Table S2 The primers used in MINI-Gene essay**

| Name | Primer sequence (5′−3′) |
| --- | --- |
| 51953-SPAST-F | GGAATGTGGACAGCAACCTT |
| 52738-SPAST-R | acttgaattctgggacgcag |
| 62021-SPAST-F | tggcgcaatcttggctcaata |
| 62356-SPAST-F | ttttgttcacgtcagcgtgc |
| 64922-SPAST-R | accttccacaactgtgagac |
| 65149-SPAST-R | gggtgggtcctaatccacag |
| pcMINI-SPAST-kpnI-F | ggtaGGTACCctcactgcacccttggcctc |
| pcMINI-SPAST-BamHI-R | TAGTGGATCCgaggtcaggagttcgacacc |
| pcDNA3.1-SPAST-KpnI-F | GCTTGGTACCatgaaTGGAACAGCTGTTAAATTTG |
| pcDNA3.1-SPAST-lapping-F | tatactaaaataattgatgctttttagatg |
| pcDNA3.1-SPAST-lapping-R | catctaaaaagcatcaattattttagtata |
| pcDNA3.1-SPAST-BamHI-R | TAGTGGATCCGTATTTTGAAGTTAAACTTG |

**Table S3 Primers for constructing the eukaryotic expression vector of SPAST**

| Name | Primer sequence (5′−3′) |
| --- | --- |
| pEGFP-C1-SPAST-HindIII-F | GCTCAAGCTTccATGAATTCTCCGGGTGGACG |
| pEGFP-C1-SPAST-BamHI-R | CGGTGGATCCAACAGTGGTATCTCCAAAGTC |
| SPAST-MUT-F | TCTCTGAGGCCTGAGGCTAAAGCAGTAGCTG |
| SPAST-MUT-R | CAGCTACTGCTTTAGCCTCAGGCCTCAGAGA |
| phage-SPAST-SalI-F | TGACGTCGACcATGAATTCTCCGGGTGGACG |
| phage-SPAST-NotI-R | CGACGCGGCCGCgAACAGTGGTATCTCCAAAGT |

**Table S4 The primers for detecting mRNA expression in 293T cells**

| Name | Primer sequence (5′−3′) |
| --- | --- |
| SPAST-phage-QPCR-F | ACGTCGACCATGAATTCTCC |
| SPAST-phage-QPCR-R | AAGCCTACAAACAGCGGGTA |
| SPAST-EGFP-QPCR-F | TCTCGAGCTCAAGCTTCCAT |
| SPAST-EGFP-QPCR-R | CCTACAAACAGCGGGTAGGA |

**Table S5 Antibody list used in this study**

| **Antibodies** | **Company** | **Catalogue No.** | **Dilution for western blot** | **Dilution for IF** |
| --- | --- | --- | --- | --- |
| **Primary Antibodies** | | | | |
| HA | Dia-an | 2063 | 1:2000 | NA |
| GAPDH | Cell signaling | 2118S | 1:1000 | NA |
| GFP | Dia-an | 2057 | 1:1000 | NA |
| acetyl-α-Tubulin | Sigma | T7451 | 1:2000 | NA |
| α-Tubulin | ABclonal | A6830 | NA | 1:100 |
| **Secondary Antibodies** | | | | |
| Anti-mouse IgG-HRP | Cell signaling | #7076 | 1:10000 | NA |
| Anti-rabbit IgG-HRP | Cell signaling | #7074 | 1:10000 | NA |
| Goat anti-Rabbit IgG, Alexa Fluor 546 | Invitrogen | A11010 | NA | 1:1000 |

*NA; not applicable
